# Multiplanar 4D strain analysis with spatial mapping to 3D LGE quantification: relationships in chronic Ischemic Cardiomyopathy

**DOI:** 10.1186/1532-429X-17-S1-P33

**Published:** 2015-02-03

**Authors:** Alessandro Satriano, Vijay Kandalam, Yoko Mikami, Nita Guron, Hanna Medwid, Bobby Heydari, Naeem Merchant, Andrew G Howarth, Carmen Lydell, Teresa A Whitman, Maria Drangova, Raymond Yee, James A White

**Affiliations:** 1 Stephenson Cardiac Imaging Centre, Libin Cardiovascular Institute, Calgary, AB, Canada; 2 Division of Cardiology, Department of Medicine, University of Calgary, Calgary, AB, Canada; 3 Department of Diagnostic Imaging, University of Calgary, Calgary, AB, Canada; 4 Queen's University, Kingston, ON, Canada; 5 Medtronic, Inc., Minneapolis, MN, USA; 6 Department of Medicine, Western University, London, ON, Canada; 7 Robarts Research Institute, Western University, London, ON, Canada

## Background

Myocardial strain analysis has been proposed as a surrogate for regional replacement fibrosis (scar) in patients with ischemic cardiomyopathy (ICM). However, contractile function is often degraded in non-scarred tissue, conceivably due to a composite of interstitial fibrosis, metabolic aberrations and abnormal electro-mechanical coupling. We tested a novel 4D strain analysis tool to examine strain characteristics of scarred and non-scarred myocardium in patients with advanced ICM.

## Methods

**Figure 1:**
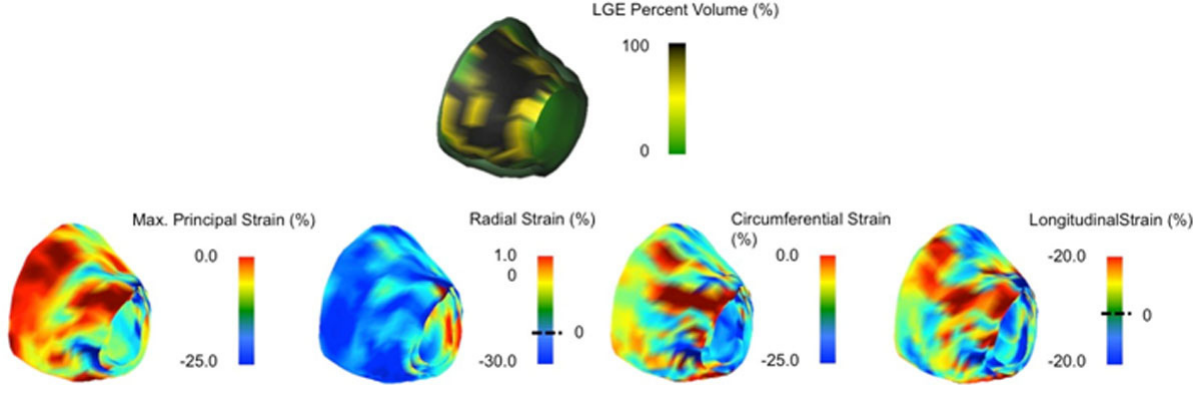
**Scar and strain LV 3D distribution.***Top Pane*: Distribution of Late Gadolinium Enhancement (Relative Enhanced Aread) across the endocardial LV surface. *Bottom Pane*: Peak-Systolic 3D distribution of Maximum Principal, Radial, Circumferential and Longitudinal Strain.

Nineteen patients with ICM and 10 healthy controls were studied. Cine and Late Gadolinium Enhancement (LGE) imaging was performed using 3.0T MRI. LV signal threshold-based (>6SD) %LGE maps were obtained using cvi42 (Circle Cardiovascular Inc., Calgary, Canada). 4D strain analysis (Figure 1) was performed using novel prototype software employing a 4D displacement field, providing spatially matched Green-Lagrange 2^nd^ principal, radial, circumferential and longitudinal strain maps. %LGE and strain were co-registered to a 72-segment model.

## Results

**Figure 2:**
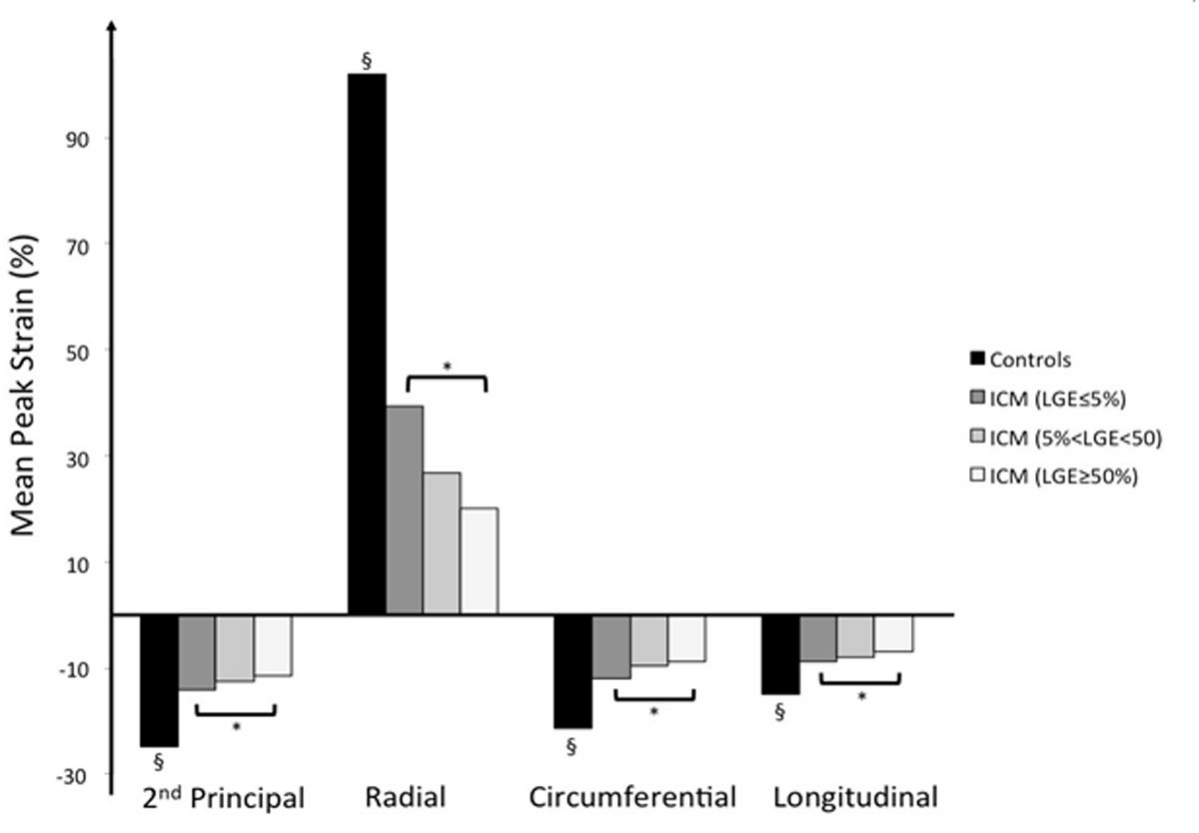
**Mean peak strain amplitude.** Mean peak strain amplitude for healthy controls and for Ischemic Cardiomyopathy (ICM) patients with no LGE (≤5%), non-transmural LGE (5%-50%),and transmural LGE (≥50%). *p<0.05 between indicated groups,§ p<0.05 versus all other groups

Mean age of ICM patients was 72.3±6.8 years with LVEF of 26.5±7.7%. Among 1368 analyzed segments, 823 had no LGE(<5%), 299 had 5-50%LGE, 246 had LGE≥50%(transmural). Mean age of healthy controls was 28.2±7.5 years with LVEF of 61.8±7.4%, all segments with no LGE. Segmental strain analysis using all 4 metrics showed substantial reductions in mean peak amplitude for ICM segments without LGE versus healthy controls (p<0.05). Within the ICM cohort, LGE≥50% segments showed reduced strain amplitudes versus segments without LGE (mean reduction 29.0±13.6% - Figure 2) for all strain metrics (p<0.05). Significant difference was found between LGE<50% and LGE≥50% segments. ROC analysis identified AUCs for detection of LGE≥50% of 0.63, 0.28, 0.62, and 0.62, respectively. Using optimal cut-offs, corresponding sensitivity was 59.8%, 32.5%, 58.5%, and 57.7%, while specificity was 59.1, 32.3%, 58.2 and 57.8%. AUCs for identifying viable (LGE<50%) segments were 0.37, 0.72, 0.38 and 0.38, the greatest sensitivity and specificity being 68.5% and 67.5%, respectively, for Radial Strain. The PPV and NPV achieved for identifying a viable segment were 90.6% and 32.0%, respectively.

## Conclusions

In chronic ICM, spatially matched 4D strain/LGE analysis identifies reduced strains in scarred segments, however also significant pathology in remote tissue compared to healthy controls. The latter limits the NPV of strain analysis for identifying non-scarred segments. However, this study demonstrates a novel capacity of CMR-based strain quantification to characterize the global health of remote tissue. As such, this provides a novel imaging marker for the quantification of remote tissue remodeling / functional integrity and warrants investigation for its prognostic value in ICM.

## Funding

Dr. Satriano receives support from Mitacs Canada and Medtronic of Canada, Ltd. Dr. White is supported by a New Investigator Award from Alberta Heart and Stroke Foundation.

